# Optimal responses in disease activity scores to treatment in rheumatoid arthritis: Is a DAS28 reduction of >1.2 sufficient?

**DOI:** 10.1186/s13075-016-1028-8

**Published:** 2016-06-16

**Authors:** Aneela N. Mian, Fowzia Ibrahim, David L. Scott, James Galloway, On behalf of the TITRATE study group

**Affiliations:** Department of Rheumatology, King’s College Hospital, Denmark Hill, London, SE5 9RS UK; Department of Rheumatology, King’s College School of Medicine, King’s College London, Weston Education Centre, Denmark Hill, London, SE5 9RT UK

**Keywords:** Rheumatoid arthritis, DAS28, Response criteria

## Abstract

**Background:**

The overall benefit of intensive treatment strategies in rheumatoid arthritis (RA) remains uncertain. We explored how reductions in disability and improvements in quality of life scores are affected by alternative assessments of reductions in disease activity scores for 28 joints (DAS28) in two trials of intensive treatment strategies in active RA.

**Methods:**

One trial (CARDERA) studied 467 patients with early active RA receiving 24 months of methotrexate monotherapy or steroid and disease-modifying anti-rheumatic drug (DMARD) combinations. The other trial (TACIT) studied 205 patients with established active RA; they received 12 months of treatment with DMARD combinations or biologic agents. We compared changes in the health assessment questionnaire (HAQ) and Euroqol-5D (EQ5D) at trial endpoints in European League Against Rheumatism (EULAR) good and moderate EULAR responders in patients in whom complete endpoint data were available.

**Results:**

In the CARDERA trial 98 patients (26 %) were good EULAR responders and 160 (32 %) were EULAR moderate responders; comparable data in TACIT were 66 (35 %) and 86 (46 %) patients. The magnitude of change in the HAQ and EQ5D was greater in both trials in EULAR good responders than in EULAR moderate responders. HAQ scores had a difference in of –0.49 (95 % CI –0.66, –0.32) in the CARDERA and –0.31 (95 % CI –0.47, –0.13) in the TACIT trial. With the EQ5D comparable differences were 0.12 (95 % CI 0.04, 0.19) and 0.15 (95 % CI 0.05, 0.25). Both exceeded minimum clinically important differences in HAQ and EQ5D scores.

**Conclusions:**

We conclude that achieving a good EULAR response with DMARDs and biologic agents in active RA results in substantially improved mean HAQ and EQ5D scores. Patients who achieve such responses should continue on treatment. However, continuing such treatment strategies is more challenging when only a moderate EULAR response is achieved. In these patients evidence of additional clinically important benefits in measures such as the HAQ should also be sought.

## Background

A key goal in treating rheumatoid arthritis (RA) with conventional disease-modifying anti-rheumatic drugs (DMARDs) and biologic agents is reducing inflammatory synovitis, which reduces disability and maximises quality of life. Reductions in synovitis with treatment are assessed using the disease activity score for 28 joints (DAS28) [1]. The European League Against Rheumatism (EULAR) classifies good responders as having DAS28 scores of 3.2 or less with reductions in DAS28 of more than 1.2 [2]. Patients with DAS28 scores over 3.2 who have reductions in DAS28 scores of more than 1.2 have moderate responses. Patients with reductions in DAS28 of less than 0.6 are non-responders. The treat-to-target initiative recommends increasing treatment until patients achieve remission or low disease activity [3].

High-cost biologic treatments such as tumour necrosis factor inhibitors are used within agreed guidelines. When patients achieve good EULAR responses there is strong rationale for maintaining treatment. Similarly, when patients do not respond there is no reason to continue treatment. However, there is uncertainty in patients with incomplete responses. The British Society for Rheumatology (BSR), recommends patients with active RA, who have not responded to methotrexate and another DMARD, should be considered for biologic therapy [4]. Provided they achieve reductions in DAS28 of over 1.2 by 6 months they should continue receiving biologic treatments. This approach was accepted by the National Institute for Health And Care Excellence (NICE) in its health technology appraisals of biologic treatments for RA [5, 6]. Initially NICE recommended only continuing treatment if DAS28 is reduced by 1.2 or more [5], though recent guidelines recommend patients achieve at least a moderate EULAR response for treatment to continue [6].

The impact on disability and quality of life of reducing disease activity are assessed using the Health Assessment Questionnaire (HAQ) [7] and EuroQol-5D (EQ5D) [8]. There are doubts about the value both to patients and the funders of healthcare from maintaining high-cost treatments if patients only achieve moderate EULAR responses. We hypothesised that patients with good EULAR responses have substantially larger improvements in HAQ and EQ5D scores than patients with moderate EULAR responses, who do not achieve low disease activity or remission, and in whom the final DAS28 scores remain over 3.2. We tested our hypothesis in secondary analyses of two published randomised controlled trials (RCTs). One trial, cost-effectiveness of treatment strategies using combination of disease modifying anti-rheumatic drugs and glucocorticoids in early rheumatoid arthritis (CARDERA), studied intensive DMARD treatment in early RA [9]. The other trial, tumour necrosis factor inhibitors against combination intensive therapy (TACIT), studied intensive treatment with DMARDs and biologic agents in established RA [10]. In both trials we compared changes in HAQ and EQ5D scores in patients achieving good EULAR responses with patients achieving reductions in DAS28 scores over 1.2, and who had only moderate EULAR responses.

## Methods

### Patients

We undertook secondary analyses using data from two completed randomised controlled trials in RA. Details of the patients and treatments used have been published [8, 9]. The CARDERA trial randomised 467 patients to monotherapy or combination therapy with conventional DMARDs and steroids in early untreated active RA. The TACIT trial randomised 205 patients to intensive conventional DMARDs or biologic treatment strategies in established active RA. In TACIT all patients met the current NICE criteria for biologic treatments.

### Assessments

Both trials used the DAS28 calculated with erythrocyte sedimentation rate (ESR) to assess disease activity. We studied patients at the predefined final endpoints of each trial: 24 months for CARDERA and 12 months for TACIT. Both trials measured disability using the HAQ and quality of life using the EQ5D. We applied reported minimal clinically important differences (MCID) to assess benefits from reducing DAS28 scores. For the HAQ we used an MCID of 0.22 [11]. For the EQ5D we used an MCID of 0.07 [12]. We used the DAS28 to assess remission, which was defined by DAS28 < 2.6; this definition has been widely used [13].

### Analyses

We used IBM SPSS statistical software (version 22). Descriptive statistics described means, standard error and confidence intervals. We studied patients with all data available at the trial endpoints. We divided patients into EULAR non-responders, moderate responders and good responders. We compared changes in HAQ and EQ5D scores between good and moderate EULAR responders for each trial using the independent samples *t* test. We also subdivided moderate and good EULAR responders by their final DAS28 scores. In addition, we used the previous NICE criterion for remaining on treatment (change in DAS28 score >1.2) to categorise patients, replicating EULAR response criteria by dividing patients into those who also achieved DAS28 low disease activity scores at the trial endpoint and those who did not. Finally, we assessed the numbers of patients who achieved different levels of improvement in HAQ and EuroQol scores in both trials in relation to moderate and good EULAR responses.

## Results

### Patients studied

In the CARDERA trial 121 patients (32 %) were EULAR non-responders, 160 (42 %) moderate responders and 98 (26 %) good responders. In the TACIT trial 34 patients (18 %) were EULAR non-responders, 86 (46 %) moderate responders and 66 (35 %) good responders. The trial designs differed, with all patients in TACIT but not CARDERA receiving intensive therapy; consequently, differences in response rates were expected. In both trials demographic characteristics, clinical variables like DAS28 scores and components of the DAS28, HAQ and EQ5D scores were similar across groups. As this secondary analysis does not assess treatment effects no comparative data between groups are presented.

### Baseline and endpoint scores

Baseline and final endpoint data for DAS28 and HAQ and EQ5D scores in both trials for different EULAR responses are shown in Table 1. In the CARDERA trial there were no significant differences between baseline scores in any EULAR responder groups. In the TACIT trial the good EULAR responders had lower baseline HAQ scores and higher baseline EQ5D scores.

**Table 1:** Baseline and endpoint assessments in completers in the TACIT and CARDERA trials (mean scores (95 % confidence intervals))

EULAR Response	Number	DAS28		HAQ		EQ5D	
		Initial	Endpoint	Initial	Endpoint	Initial	Endpoint
CARDERA trial							
None	121	5.45 (5.22, 5.68)	5.67 (5.45, 5.89)	1.43 (1.30, 1.56	1.53 (1.39, 1.67)	0.46 (0.40, 0.51)	0.48 (0.43, 0.53)
Moderate	160	6.08 (5.89, 6.27)	4.21 (4.07, 4.35)	1.70 (1.60, 1.80)	1.31 (1.20, 1.42)	0.42 (0.37, 0.46)	0.60 (0.56, 0.63)
Good	98	5.57 (5.33, 5.80)	2.17 (2.04, 2.30)	1.47 (1.35, 1.60)	0.60 (0.48, 0.72)	0.47 (0.42, 0.52)	0.77 (0.73, 0.80)
TACIT trial							
None	34	6.40 (6.08, 6.71)	6.34 (6.06, 6.63)	2.15 (2.00, 2.29)	2.11 (1.94, 2.28)	0.22 (0.11, 0.33)	0.23 (0.13, 0.34)
Moderate	86	6.30 (6.11, 6.49)	4.23 (4.07, 4.38)	1.91 (1.78, 2.04)	1.58 (1.42, 1.73)	0.41 (0.35, 0.47)	0.56 (0.51, 0.61)
Good	66	6.13 (5.96, 6.30)	2.29 (2.14, 2.44)	1.64 (1.47, 1.81)	1.00 (0.81, 1.19)	0.40 (0.32, 0.47)	0.69 (0.64, 0.75)

*TACIT* tumour necrosis factor inhibitors against combination intensive therapy, *CARDERA* cost-effectiveness of treatment strategies using combination of disease modifying anti-rheumatic drugs and glucocorticoids in early rheumatoid arthritis, *EULAR* European League Against Rheumatism, *DAS28* twenty-eight joint disease activity score, *HAQ* Health Assessment Questionnaire, *EQ5D* Euroqol-5D

### Differences in disability between EULAR moderate and good responders

Only patients with either moderate or good EULAR responses had significant reductions in HAQ scores at the trial endpoints (Table 2). Moderate responders had reductions in HAQ scores of 0.39 and 0.33 in the CARDERA and TACIT trials, respectively. Good responders had reductions of 0.88 and 0.64, respectively. In both trials the difference between moderate and good responders exceeded the MCID for HAQ scores (0.22) with differences in reductions of 0.49 and 0.30, respectively. These differences were significant (*p* < 0.01, unpaired *t* test).

**Table 2:** Changes in disability (HAQ scores) by EULAR response mean scores (95 % confidence intervals)

	None	Moderate	Good	Difference moderate and good
CARDERA	0.10 (–0.01, 0.21)	–0.39 (–0.48, –0.30)	–0.88 (–1.02, –0.73)	–0.49 (–0.66, –0.32)
TACIT	–0.04 (–0.15, 0.08)	–0.33 (-0.44, -0.22)	–0.64 (–0.77, –0.51)	–0.30 (–0.47, –0.13)

*EULAR* European League Against Rheumatism, *CARDERA* cost-effectiveness of treatment strategies using combination of disease-modifying anti-rheumatic drugs and glucocorticoids in early rheumatoid arthritis, *TACIT* tumour necrosis factor inhibitors against combination intensive therapy

### Changes in quality of life

The mean EQ5D scores had a similar pattern. Only patients with moderate or good EULAR responses had significant improvements in EQ5D scores at the trial endpoints (Table 3). Moderate responders had increases in EQ5D scores of 0.18 and 0.15 in the CARDERA and TACIT trials, respectively. Good responders had increases of 0.30 in both trials. The difference between moderate and good responders exceeded the EQ5D MCID (0.07) in both trials with differences in increases in EQ5D scores of 0.12 and 0.15, respectively. These differences were significant (*p* < 0.01, unpaired *t* test).

**Table 3:** Changes in health-related quality of life (EQ5D scores) by EULAR response mean scores (95 % confidence intervals)

	None	Moderate	Good	Difference moderate and good
CARDERA	0.02 (–0.03, 0.07)	0.18 (0.14, 0.23)	0.30 (0.24, 0.35)	0.12 (0.04, 0.19)
TACIT	0.00 (–0.13, 0.14)	0.15 (0.08, 0.21)	0.30 (0.22, 0.37)	0.15 (0.05, 0.25)

*EQ5D* EuroQol-5D, *EULAR* European League Against Rheumatism, *CARDERA* cost-effectiveness of treatment strategies using combination of disease-modifying anti-rheumatic drugs and glucocorticoids in early rheumatoid arthritis, *TACIT* tumour necrosis factor inhibitors against combination intensive therapy

### Alternative assessments of response

Similar analyses based on patients achieving reductions in DAS28 > 1.2, subdivided by whether or not they also achieved low DAS28 at the trial endpoints, were similar in relation to both HAQ and EQ5D scores. Patients who did not achieve low disease activity scores had only modest improvements in both scores (detailed results not shown).

### Additional benefits of achieving remission

Another analysis subdivided patients achieving good EULAR responses into those who were also in remission and those who were not at the trial endpoints. In the CARDERA trial 70/98 patients (71 %) with good responses were in remission and 28/98 (29 %) were not. There were non-significant benefits for both HAQ scores and EQ5D scores with remission; mean differences between groups were –0.29 (95 % CI –0.61, 0.02) for HAQ scores and 0.11 (–0.02, 0.24) for EQ5D scores. In the TACIT trial 41/66 patients (62 %) with good responses were in remission and 25/66 (38 %) were not. There were also non-significant benefits for both HAQ scores and EQ5D scores with remission. The mean differences between the groups were –0.13 (–0.41, 0.14) for HAQ and 0.08 (–0.07, 0.24) for EQ5D.

### Categories of HAQ and EQ5D change with EULAR responses

**Fig. 1:**
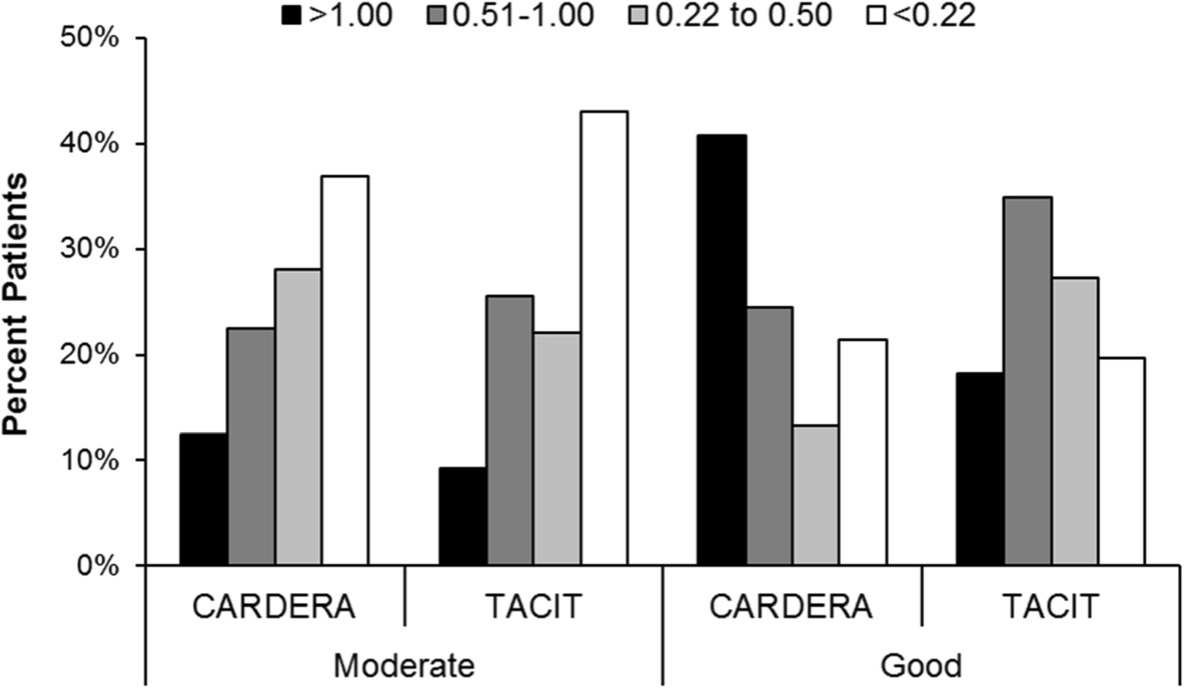
Changes in Health Assessment Questionnaire (*HAQ*) scores in four categories in moderate and good European League Against Rheumatism (EULAR) responders in both the cost-effectiveness of treatment strategies using combination of disease-modifying anti-rheumatic drugs and glucocorticoids in early rheumatoid arthritis (*CARDERA*), and tumour necrosis factor inhibitors against combination intensive therapy (*TACIT*) trials

In the CARDERA trial 63 % of patients with moderate EULAR responses had improvements in HAQ exceeding the MCID (0.22) compared with 79 % of patients with good EULAR responses. In the TACIT trial 57 % of patients with moderate EULAR responses had improvements in HAQ exceeding the MCID compared with 80 % of patients with good EULAR responses. On more detailed analysis (Fig. 1) there was a wide range in HAQ score improvements in both trials in moderate and good EULAR responders; more patients with large improvements in HAQ scores exceeding 1.0 had good EULAR responses, particularly in the early RA trial (CARDERA). In this trial 41 % of patients with good EULAR responses had improvements in HAQ over 1.0.

**Fig. 2:**
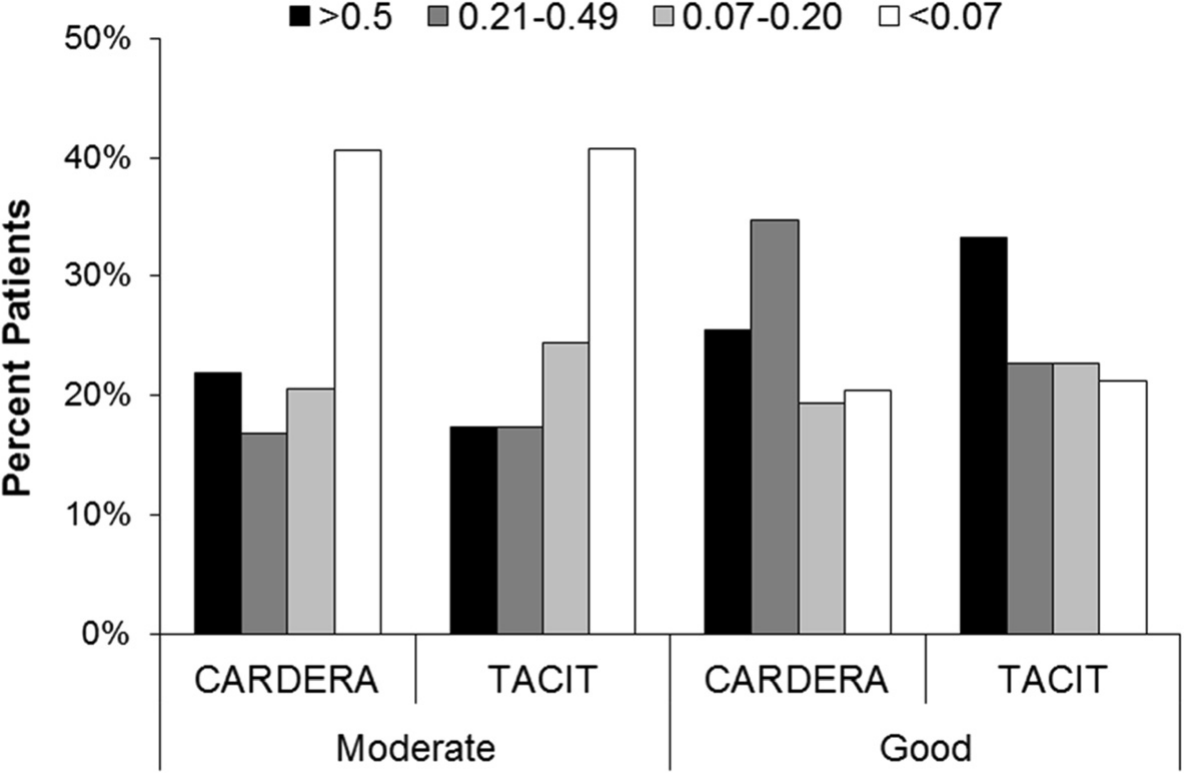
Changes in EuroQol-5D (EQ5D) scores in four categories in moderate and good European League Against Rheumatism (EULAR) responders in both the cost-effectiveness of treatment strategies using combination of disease-modifying anti-rheumatic drugs and glucocorticoids in early rheumatoid arthritis (*CARDERA*), and tumour necrosis factor inhibitors against combination intensive therapy (*TACIT*) trials

Changes were similar with the EQ5D scores. In the CARDERA trial 59 % of patients with moderate EULAR responses had improvements in EQ5D scores exceeding the MCID (0.07) compared with 80 % of patients with good EULAR responses. In the TACIT trial 59 % of patients with moderate EULAR responses had improvements in EQ5D scores exceeding the MCID compared with 79 % of patients with good EULAR responses. On more detailed analysis (Fig. 2) there was a wide range in improvement in the EQ5D scores in both trials in moderate and good EULAR responders; more patients with good EULAR responses had large improvements in EQ5D scores. The benefits from achieving good EULAR responses were less marked with improvements in EQ5D scores.

## Discussion

Our secondary analyses of two large English RCTs in early and established active RA shows that patients with good EULAR responses have larger clinically meaningful improvements in disability and quality of life than patients with moderate EULAR responses. These findings support the concept of treat-to-target. They indicate that achieving low disease activity or remission states should be the treatment goal. The benefits of good EULAR responses are seen in both early and established RA. The findings were similar if we used the earlier NICE criterion of an improvement in DAS28 of more than 1.2.

The reliance of current NICE guidance on changes in DAS28 of more than 1.2 has been criticised by Jerram et al [14], who point out that this threshold was not used in clinical trials. This concern is ameliorated by the new NICE guidance. Judging patients’ responses to DMARDs and biologic therapy is challenging. EULAR response criteria were designed for use in individual patients and trials. The other major composite response criteria, the American College of Rheumatology (ACR) criteria, are only intended for trials. These two validated response criteria have been compared in several studies [15, 16]; they measure similar changes. However, their use in routine clinical practice and relating their findings to patients’ perceived responses requires more research. Gulfe et al. showed that when response criteria are used at the individual patient level the results are difficult to interpret [17]. Ward et al. [18] found patients’ perceptions of improvement may differ from conventional assessments like ACR20 responses, and that thresholds of minimal clinically important improvement may be larger than previously thought, including the need for a DAS28 improvement of at least 1.2 [19]. Among patients receiving treatment for active RA, three months may be the optimal time point to increase the intensity of treatment [20]. However, when assessing response to biologic therapy, real-world evidence suggests a six-month time point is needed to optimise patients’ responses [21].

Low disease activity optimises key patient-related outcomes like HAQ and EQ5D scores [22–24]. Our own findings mirror these previous reports. Remission is one potentially important outcome, although only a minority of our patients achieved this. Interestingly some other trials in early RA, such as the FIN-RACo trial [25], reported higher remission rates; the reasons for this heterogeneity are uncertain. When patients achieve sustained remission, they have less long-term work disability [26]. Some patients with moderate EULAR responses in our trials had good reductions in disability and improvements in quality of life. However, many did not and the best management of such patients is uncertain. Continuing to prescribe high-cost biologic therapy for patients with modest improvements in disease activity and little or no reduction in disability seems questionable. It may be preferable to continue biologic treatments in patients with moderate EULAR responses who have also had clinically meaningful reductions in disability.

Our report has several strengths. The two trials were large, there was reasonable patient retention on treatment, and they were based across many English specialist centres with a broad geographic spread. We found similar effects in trials of early and established RA. Our findings are therefore likely to be generalisable in guiding clinical practice decisions.

Our analyses also have limitations. First, we only considered differences at the trial endpoints. Different effects would have been seen by evaluating all time points. Second, choosing specific cutoff points, such as DAS28 of 3.2 or less may have over done similarly well. Third, some of the treatments used, particularly ciclosporin, which was used in the CARDERA trial, are not commonly prescribed in routine practice. Fourth, we combined the effects of a range of different treatments, and the relationship between disease activity and HAQ and EQ5D scores may be different across drug classes. Finally, our inference that patients who do not achieve low DAS28 scores with one treatment strategy may do better with another could be incorrect; some patients may achieve poor outcomes with all treatment strategies.

## Conclusions

Achieving good EULAR responses to treatment with DMARDs and biologic agents leads to substantial improvements in HAQ and EQ5D scores. Patients who achieve such responses should continue on treatment. However, continuing patients on intensive treatment regimens, particularly using high-cost biologic agents, is more challenging if they only achieve moderate EULAR responses. Evidence of additional clinically important benefits in measures such as the HAQ should also be sought to justify continuing treatment in these patients.

## Abbreviations

ACR, American College of Rheumatology; CARDERA, Cost-effectiveness of treatment strategies using combination of disease-modifying anti-rheumatic drugs and glucocorticoids in early rheumatoid arthritis; CI, confidence interval; DAS28, twenty-eight joint disease activity score; DMARD, disease-modifying anti-rheumatic drug; EQ5D, Euroqol-5D (a standardized instrument for measuring generic health status); EULAR, European League Against Rheumatism; HAQ, Health Assessment Questionnaire; MCID, minimal clinically important difference; NICE, National Institute of Health and Care Excellence; RA, rheumatoid arthritis; RCT, randomized controlled trial; TACIT, tumour necrosis factor inhibitors against combination intensive therapy
